# Suffering London: The "Globe's" Parable

**Published:** 1892-06-04

**Authors:** 


*For Hospitals, Asylums, Infirmaries, Dispensaries, Medical Practitioners, Students, Nurses, and the Charitable Public.*

VOL. XII.—No. 297.] [JUNE 4, 1892.

### SUFFERING LONDON: THE "GLOBE'S" PARABLE.

ON the subject of the great concerted effort which the metropolitan voluntary hospitals are now uniting to put forth, the *Globe*, of May 28th, made the following terse and apposite criticisms, which we quote in full: "On behalf of a book entitled 'Suffering London,' which Mr. Egmont Hake has written, and for which Mr. Walter Besant has furnished an introduction, the Editor of THE HOSPITAL makes a special appeal to the Press. The newspapers were ready enough, he says, to back up Mr. Booth's 'Darkest England' scheme; but he implies that they are less ready to recognise the value of hospitals and to urge their claims upon the attention and generosity of the public. We venture to think that, in most cases at any rate, neither of these statements is strictly accurate. The Press, as a whole, did not back up Mr. Booth's scheme on the one hand; on the other, it is fully alive to the importance of hospitals and the necessity for their support. The 'Darkest England' business, of course, received the attention which in these days is accorded to everything that is new and startling; but the hospitals in the course of the year receive a far larger amount of public notice, and of a less equivocal kind. Hospital Sunday, which will soon be upon us again, every year forms the subject of innumerable leading articles, which are practically all sympathetic in tone. In fact, it is difficult to say more in favour of hospitals than has been said hundreds of times before. One difficulty in the way is the attitude of the hospitals themselves. When we are asked to assist 'the uplifting and permanent establishment of one of the greatest and noblest and most absolutely indispensable "causes of modern times,"' we are fain to enquire what is meant by 'permanent establishment?' If it means a larger generosity in the matter of 'voluntary contributions,' then we are ready to do as we have always done in bringing the claims of the hospitals before the public. But if, as rather seems to be intended, it means 'State aid,' then the matter becomes more difficult. For 'State aid' implies 'State control,' and we very much doubt whether the hospitals are prepared, even for the sake of financial stability, to surrender their independence."

This article is quoted in full because it expresses, probably, what a great many newspaper writers are feeling, and what a great many readers of newspapers are feeling as well. It is clear, from the general tone of the criticism, that the *Globe*, is most friendly; and we fully believe that most of the metropolitan newspapers are friendly too. What they seem to say to us who venture to speak on behalf of the hospitals is this: "We are doing all we can for you; we have long done all we could—why are you dissatisfied? What more do you want"? If this be the present attitude of the Press, we have got at least one thing we wanted—we wanted to be asked those very questions which we have supposed the Press to be asking us.

Let us now take the last of the difficulties which the *Globe* sets forth in its article. What do you mean, says the enquirer, by the "permanent establishment" of the metropolitan and country hospitals? Do you mean that they ought to be supported wholly or in part by the State? Most emphatically we do not; and we are convinced that there is not a successful hospital in London or the country which does. What do we mean then? A line of the Laureate's occurs to us which well expresses what the most enlightened hospital statesmen would fain be at. The Laureate tells us that the throne of Queen Victoria is "Broad based upon the people's will." Now that is exactly what the hospitals are not. They are narrow based; based upon the will of a few. The few are so very few that the hospitals cannot be maintained at their present rate of work. But their present rate of work is in many important particulars inadequate. If then the needs of the needy have already very considerably outrun the resources of the philanthropic, what is the prospect in the immediate future for a rapidly-growing population?

The statesmen who have devoted their best energies to hospitals and charities rather than to local or imperial politics, have long felt that the hospital system has been assuming the form of a pyramid, and of a pyramid balanced on its apex. What they feel they must do is to turn the pyramid upside down, and to plant it securely on its base: or, better still, to buttress the apex on both sides with such solid and wide-extending masonry that the pyramid shall be mightily enlarged, and its apex converted into its all-sufficient base. The first thing, then, that hospital statesmen ask of a friendly press is its enthusiastic co-operation in a united effort to broaden the basis of hospital support. The hospital system, as we have maintained, is an indispensable branch of the public service. This view of the case we desire to see clearly recognised. But if the fact be so, then the whole public, and not a mere few, ought to support hospitals. We are probably well within the fact when we state that not more than one adult person in fifty gives anything systematically to hospitals, and probably not more than one in ten gives anything at all. But is this the way to support a great public service? A public service which is beneficial to all classes, and to all classes in almost equal proportions? We most gratefully admit the help of the press in constantly reporting the proceedings of separate hospitals, and in making periodical appeals for the institutions in the mass. But a crisis has now been reached in hospital affairs; and the crisis is of such a kind that it can be dealt with in no other way but by the better instruction of the public conscience and judgment as a whole.

The real difficulty lies in the fact that hospitals are voluntary institutions. But this, which is their difficulty, is also their crowning glory. If hospitals were State institutions the appeal for their broader maintenance would necessarily be to Parliament; but being institutions of a voluntary and popular character the appeal must as inevitably be to the people. What we contend for, with even wearisome iteration, and what we are anxious that the entire press should contend for, is that the appeal must be to the whole people, and not any longer to the few. The appeal, moreover, must be to reason, to intelligence, and even to enlightened selfishness, as well as to philanthropy and religion. Philanthropy and religion have hitherto borne the brunt of the battle. But they cannot bear it any longer: they are being defeated at many points; and though they will never cease to fight, they are being compelled to fight a losing battle. Now the public cannot afford to let them fight a losing battle: and that is why we insist that the general reason, and the general intellect, and the enlightened selfishness of those who care nothing for philanthropy should be appealed to and instructed in this great public question.

It is imperative (as a preliminary) that the whole people, and not merely the few, should recognise the value of hospitals; their value to the State as a State, to towns and cities as towns and cities, and to classes and individuals as classes and individuals. If this be once recognised, the task of raising money, which now appears gigantic and impossible, will become mere child's play. Some persons who have never studied the question, think it very unlikely that we shall be able during a long future to carry on the hospitals of our country on a purely voluntary basis. We differ from those persons entirely, and that on three especial grounds: because, first, we believe in the progress of man; because, secondly, we believe still more in the progress of the English man; and because, in the third place, we believe most of all in the progress of religion and a reasonable Christianity. If we want to know what voluntary effort can do in a trusted cause, we have but to look at the history of the English nation, and at the history of the religion of the English nation. The English people have spread themselves over the entire world, not at the bidding of imperial conquerors, but in obedience to the mandate of their individual resolution. Similarly the religion of England, both in and out of the National Church, has advanced from victory so victory, and from conquest to conquest, ever tince it learnt to trust its appeal to the good sense and good will of a generous people. Convert the whole public to an intelligent appreciation of the imperial, the municipal, and the personal value of hospitals; and show them that of all hospitals those that are supported by voluntary gifts are the kindliest, the gentlest, the most scientific, and the most effective for their purpose, and you will by a single stroke permanently establish the hospital system on a foundation that can never be moved.

